# Incidence Rates of Melanoma and Lung Cancer Are Generally Low in the Lynch Syndromes and Vary Across *path_MMR* Variants: A Prospective Lynch Syndrome Database Report

**DOI:** 10.3390/cancers18132177

**Published:** 2026-07-07

**Authors:** John D. Potter, Finlay A. Macrae, Julian R. Sampson, Saskia Haupt, Toni T. Seppälä, Sinead Cameron-Mackintosh, Aysel Ahadova, Matthias Kloor, Pål Møller, PLSD Collaborators

**Affiliations:** 1 Public Health Sciences, Fred Hutchinson Cancer Center, Seattle, WA 98109, USA; 2 Department of Epidemiology, University of Washington, Seattle, WA 98195, USA; 3 Centre for Public Health Research, Massey University, Wellington 6140, New Zealand; 4 Department of Medicine, University of Melbourne, Melbourne 3010, Australia; finlay.macrea@mh.org.au; 5 Division of Cancer and Genetics, Cardiff University School of Medicine, Heath Park, Cardiff CF14 4XN, UK; sampson@cardiff.ac.uk; 6 Independent Researcher, 69120 Heidelberg, Germany; 7 Faculty of Medicine and Health Technology, Tampere University and Tays Cancer Centre, Tampere University Hospital, 33521 Tampere, Finland; 8 Simply Genetics, Hilton P.O. Box 3245, South Africa; cameronmackintoshs@gmail.com; 9 Department of Applied Tumour Biology, Institute of Pathology, Heidelberg University Hospital, 69120 Heidelberg, Germany; 10 Department of Tumour Biology, Institute of Cancer Research, The Norwegian Radium Hospital, 0424 Oslo, Norway; moller.pal@gmail.com

**Keywords:** Lynch syndromes, melanoma, lung cancer, DNA mismatch repair deficiency, immune system

## Abstract

**Background/Objectives** It is not established whether melanoma and lung cancer are part of the tumor spectrum of the Lynch syndromes (LS). Our first hypothesis was that, if melanoma is associated with LS, most prospectively observed cases would be carriers of pathogenic MSH2 variants (*path_MSH2*), as for other LS non-endodermal tumors. Our second hypothesis, which was derived from findings of the first study, was that the pattern of differences in the incidence of lung cancer in *path_MMR *carriers would mirror that for melanoma. **Methods** We used the Prospective Lynch Syndrome Database (PLSD) data and methods. **Results** Firstly, consistent with hypothesis, ten of 3154 *path_MSH2* carriers followed for 26,309 years developed melanoma, compared with 6 of 5288 *path_MLH1/MSH6/PMS2* carriers followed for 45,081 years (*p* = 0.04). Secondly, although the incidence of melanoma in carriers of *path_MSH2* was 0.00041—similar to the populations in which the carriers resided—the average incidence rates for *path_MLH1/MSH6/PMS2* carriers was 0.00012, **lower** than the corresponding population rates. Thirdly, the average lung cancer incidence rates in PLSD were 0.00112 in *path_MSH2* carriers and 0.00032 in *path_MLH1/MSH6/PMS2* carriers (*p* = 0.02), both **lower** than the population rates. **Conclusions** Melanomas and lung cancers—both of which are immunogenic through mechanisms independent of mismatch repair deficiency—may become even more immunogenic when they also exhibit microsatellite instability. This may lead to an increased likelihood that both tumors are eliminated by the immune system and, thus, show variable and lower incidence. These insights may help inform strategies for cancer prevention or treatment that are aimed at simultaneously interrupting multiple carcinogenic pathways.

## Simple Summary

Stimulated by a report of melanoma in the eye of a person with one of the Lynch syndromes (LS), which cause an inherited risk of cancer, we explored whether people with LS are generally at increased risk of melanoma. Our hypothesis was that if melanoma is associated with LS, those affected would have a damaged version of one particular gene: *path_MSH2*. Consistent with this, there was a higher risk among these people than among individuals with damaged versions of the three other LS genes. However, we also found that the overall occurrence of melanoma was not higher than in the general population. Because both melanoma and lung cancer respond to immunotherapy and because the abnormal proteins in individuals with LS produce immune reactions, we then hypothesized that the pattern of lung cancer in people with LS would mirror what we found for melanoma. This was confirmed: the average lung cancer incidence rates were **lower** in people with LS than in the general population. We interpret our new findings to indicate that melanomas and lung cancers are more likely to be eliminated by the immune system among people with LS, which may help inform strategies for cancer prevention or treatment.

## 1. Background

Each of the four Lynch syndromes (LS) is an autosomal dominant trait in which cancers that show microsatellite instability (MSI) are associated with pathogenic versions of each of the DNA mismatch repair genes, namely *path_MSH2, MLH1, MSH6* and *PMS2* [1]. They are characterized by a dynamic balance between two stochastic processes: (i) mismatch repair deficiency (MMRd), which results in abnormal cells; and (ii) the capacity of the host’s adaptive immune system to eliminate these cells, which is related to the ability of infiltrating T-cells [2,3] to recognize the abnormal peptides (the neoantigen load) created by the MMRd [4,5,6,7].

The four Lynch syndromes (LS) have different penetrances and expressivities (www.plsd.eu) [1]. With three known exceptions, LS tumours arise in tissues that take their embryonic origin from the endoderm [1]. The exceptions are: (i) brain tumours, 68% of which occur in *path*_*MSH2* carriers [8]; (ii) osteosarcomas, again found mainly in *path_MSH2* carriers [9]; and (iii) sebaceous gland adenomas (the Muir–Torre variant of Lynch syndrome [10]), which also occur predominantly in *path_MSH2* carriers [11] and only rarely become malignant [12].

Embryonic ectoderm differentiates into the epidermis (giving rise to the skin, hair, nails, etc.), the neural tube (the origin of the central nervous system), and the neural crest cells, which give rise not only to the peripheral and enteric nervous systems but also to melanocytes. Stimulated by a report of a uveal melanoma in a *path_MLH1* carrier, we sought to test the hypothesis that melanoma is part of the LS tumour spectrum [13].

## 2. Methods

The Prospective Lynch Syndrome Database (PLSD) method [14] was designed to validate *a priori* substantiated theories as we report here, whereas fishing for random associations within the recorded data risks false results and is not done.

Similar to all PLSD reports, the results reflect events in carriers subjected to follow-up. Methods are detailed and discussed in previous papers. In short, empirical, assumption-free observations on all carriers who have been subjected to colonoscopy and screening for endometrial cancer at collaborating centres world-wide, were filed by observed *path_MMR* variant, sex, and cancer diagnosis (first three ICD9 digits); each cancer is considered a discrete event in time with age filed as an integer and analyzed assuming Poisson distributions. Follow-up for the first cancer ended at the first cancer; follow-up for survival ended at the last observation or death, whichever came first. All reported results are left-censored at 25 yrs or at the start of follow-up and right-censored at 75 years or at the first event, last observation, or death as appropriate. We denote all pathogenic variants of the same gene as ‘variant’, not ‘gene’.

We restricted our calculations to the time to first melanoma in carriers aged 25 to 74 years. In keeping with the usual way of calculating the incidence of events in groups (www.plsd.eu), we used the number of observed cases divided by the total number of observation years. In addition, we calculated the average incidence in each of the ten 5-year cohorts from 25 to 74 years and then averaged those, which we denote as the “average incidence rate 25–74 years”; we compared this with age-standardized rates for corresponding ages in the populations in which they occurred. This way of calculating average incidence rates adjusts for unequal numbers of cases observed in the different 5-year age groups [14].

### 2.1. Study 1

We reasoned that, if melanoma is indeed part of the LS spectrum, carriers of *path_MSH2* variants would show the highest incidence of these tumours, as is seen with other LS tumours that have non-endodermal origin. This hypothesis was based on our own and others’ findings described above and in marked contrast to the finding that only 1 of 8 uveal melanoma cases [13] in the paper stimulating our study was in a *path_MSH2* carrier. We began with a hypothesis that *path_MLH1/MSH6/PMS2* carriers would not have a low incidence of melanoma and that, if *path_MSH2* carriers had a higher incidence than other carriers, this would show that melanoma is part of LS. We specifically hypothesized further that, because there is selection bias in the way in which individuals are initially added to PLSD (based on whether or not they had been genetically tested), prospective data would provide a more reliable estimate than retrospective data.

We conducted this study using PLSD version 5 data (www.plsd.eu). Carriers who had melanoma before or at inclusion in PLSD were excluded [14,15]. Because retrospective studies have not indicated that melanoma is part of the Lynch syndromes or associated with *path_MSH2* (plausibly because the variable likelihood of testing individuals for carrier status introduces selection bias), we did a retrospective analysis of the genotypes of *path_MMR* carriers with melanomas prior to or at inclusion to compare with the prospective findings. PLSD records only the first three digits of ICD9 codes; therefore, we could not investigate the anatomic location of melanomas to compare our findings with the report of uveal melanoma in LS [13] that prompted this study.

In order to establish the stability of the findings within the PLSD, we repeated the analysis with data from the very first version of the database.

### 2.2. Study 2

We explored whether melanoma in general was increased in *path_MMR* carriers. The European Cancer Information System now offers a convenient tool (https://ecis.jrc.ec.europa.eu/data-explorer#/estimates, accessed on 30 June 2026) for calculating average incidence rates of cancer in each member country. We compared the observed overall average incidence of melanoma in study 1 with the overall incidence in Europe and compared the observed incidence in our cohort of carriers by country with the corresponding reported incidence in the populations in which they resided. As further contrasts, we used our previous report on osteosarcoma in LS and here report the incidence of brain tumours in our cohort of *path_MMR* carriers.

### 2.3. Study 3

Based (i) on our findings in studies 1 and 2 and (ii) the observation that, like MMRd LS cancers [16,17], MMR-proficient melanomas respond to immunotherapy [18], we hypothesized that lung cancer, which also responds to immunotherapy [19], would show a lower incidence in LS and variation in incidence among *path_MMR* carriers similar to our observations for melanoma.

## 3. Results

### 3.1. Study 1

**Figure 1:**
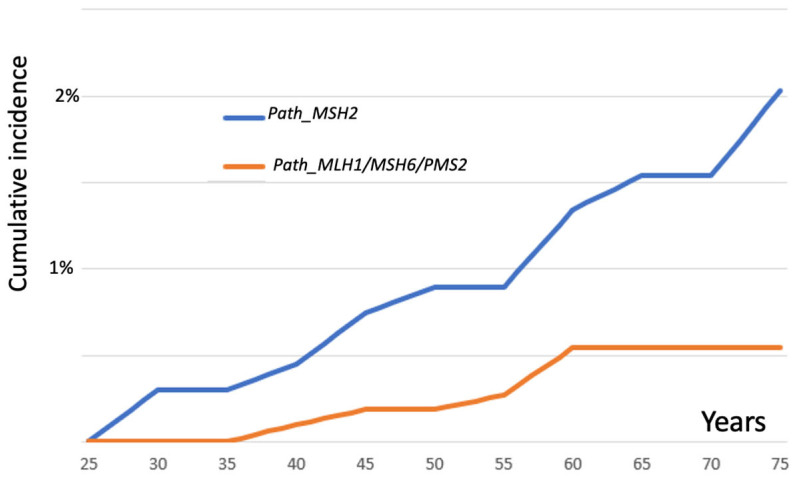
Cumulative incidence of melanoma in *path_MSH2* and *path MLH1/MSH6/PMS2* carriers from age 25 to74 years.

Among 8442 *path_MMR* carriers aged 25 to 74 years without melanoma before or at inclusion and followed for a total of 71,390 years, 16 melanomas (16/71,390 = 0.00022) were diagnosed prospectively. None of the carriers had more than one prospectively detected melanoma. The average incidence rate was 0.00041 for *path_MSH2* carriers and 0.00012 for *path_MLH1/MSH6/PMS2* carriers grouped together. The mean age at diagnosis of melanoma was 52.4 years for *path_MSH2* carriers and 51.8 years for *path_MLH1/MSH6/PMS2* carriers. In 3154 *path_MSH2* carriers aged 25–74 years who were followed for a total of 26,309 years, 10 were diagnosed with melanoma. In 5288 *path_MLH1/MSH6/PMS2* carriers followed for 45,081 years, 6 were diagnosed with melanoma (Fisher’s exact *p* = 0.04). As hypothesized, *path_MSH2* carriers had a higher incidence of prospectively diagnosed melanoma than other *path_MMR* carriers. Figure 1 shows the cumulative incidence of melanoma for *path_MSH2* and *path_MLH1/MSH6/PMS2* carriers.

In contrast to the prospective findings, retrospective data revealed 31 melanomas that had been diagnosed before or at inclusion for follow-up. Only 6/31 were in *path_MSH2* carriers compared to 10 of 16 in prospectively detected cases (Fisher’s exact *p* = 0.008).

Ten-year overall survival after prospectively diagnosed melanoma before 65 years of age was 76% (95% CI 42–92%) and did not differ between *path_MSH2* carriers and carriers of pathogenic variants of other MMR genes.

In the first dataset published by PLSD, the number of carriers and observation years was only one-third of those included in the current (version 5) dataset on which the above results are based. A separate analysis of this first, smaller PLSD dataset revealed a total of five prospectively diagnosed melanomas, of which four were in *path_MSH2* carriers, indicating that the findings in the current dataset were not caused by random variation in a single dataset.

### 3.2. Study 2

As reported above, the overall incidence of melanoma in our study was 0.00022, which is similar to the European average of 0.00024 for 2024. The incidence of melanoma has increased over the period of our prospective observations [20]. Our results might therefore indicate a slightly higher overall incidence. We do not have population data for the observation period for all countries in which the carriers lived and certainly insufficient data to partition into the 89 sub-groups of carriers by genetic variants by country of residence. However, our initial hypothesis that *path_MLH1/MSH6/PMS2* carriers would not have a lower incidence of melanoma than the general population proved wrong: *path_MLH1/MSH6/PMS2* carriers had an overall low incidence of 0.00012. Our cohort of *path_MSH2* carriers had a prospectively observed incidence of 0.00041, which suggests a higher incidence over the observation period than the population rates.

Hence, melanoma incidence, in contrast to our initial assumption, was lower than in the general population among *path_MLH1/MSH6/PMS2* carriers, whereas the incidence was possibly slightly higher among *path_MSH2* carriers than in the population.

The average incidence of brain tumours in all carriers aged 25–74 years was 0.00047: 0.00065 in *path_MSH2* and 0.00020 in *path_MLH1/MSH6/PMS2* carriers. All rates were higher than the average 0.00010 (range 0.00007–0.00012) for European countries.

In sum, we confirmed the higher incidence of brain tumours in the LS reported by others [8], whereas we found the incidence of melanoma to be low in *path_MLH1/MSH6/PMS2* carriers and possibly slightly higher than the population in *path_MSH2* carriers.

### 3.3. Study 3

Among 8437 *path_MMR* carriers aged 25 to 74 years without lung cancer before or at inclusion and followed for a total of 71,584 years, 19 lung cancers were diagnosed prospectively, giving an average incidence rate of 0.00034. Both of these values are lower than in any European country.

Among *path_MSH2* carriers followed for 26,377 observation years, 12 lung cancers were observed. Among *path_MLH1/MSH6/PMS2* carriers followed for 45,207 observation years, there were seven lung cancer cases (Fisher’s exact *p* = 0.03).

No lung cancer was observed in any *path_MMR* carrier before 50 years of age; this, in theory, might reflect changes in smoking habits over recent decades, as it has been reported that carriers under surveillance have lower rates of smoking than in the population as a whole [21]. Therefore, we also calculated annual incidence in carriers aged 50–74 years: in 33,823 observation years of 4809 carriers, 19 lung cancers were observed, giving an average incidence rate of 0.00068, lower than in any European country.

**Figure 2:**
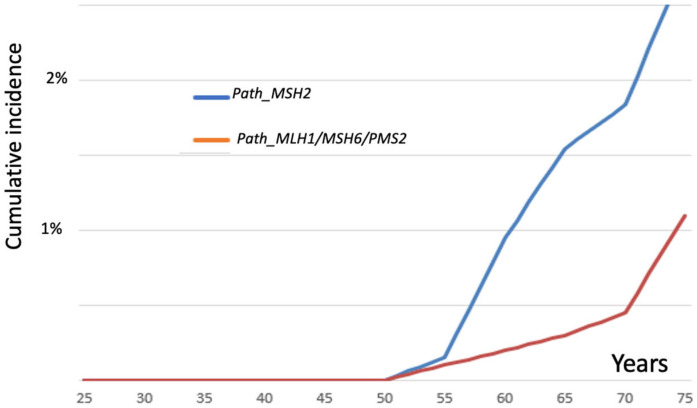
Cumulative incidence of lung cancer in *path_MSH2* and *path_MLH1/MSH6/PMS2* carriers from age 25 to 74 years.

Among 3153 *path_MSH2* carriers, 12 lung cancers were observed in 12,144 observation years, an average incidence rate of 0.00112. This contrasts with seven in 21,679 observation years in *path_MLH1/MSH6/PMS2* carriers, an average incidence rate of 0.00022 (Fisher’s exact *p* = 0.02). These values are lower than in any European country. Figure 2 shows the cumulative incidence of lung cancer for *path_MSH2* and *path_MLH1/MSH6/PMS2* carriers.

Hence, the incidence of lung cancer varied across *path_MMR* carriers, with *path_MSH2* carriers having the highest incidence and all carriers having a lower incidence than in the populations in which they resided.

The combined results of our past and present studies are that osteosarcoma and brain tumours have a higher incidence in *path_MMR* carriers than in the populations in which they live and are part of LS, whereas melanoma and lung cancer are not part of the Lynch syndromes.

Based on the conclusions above, it would be reasonable to examine whether or not the incidence of melanoma and lung cancer varied across *path_MLH1*, *path_MSH6* and *path_PMS2* carriers. In *path_MLH1* carriers aged 25–74 years observed for 31,127 years, we observed three melanoma cases, compared to three cases in *path_MSH6/PMS2* carriers with a combined 13,954 years of observation (*p* > 0.1). Correspondingly, among *path_MLH1* carriers aged 50–74 years observed for 13,422 years, three lung cancer cases were diagnosed, compared to four cases in *path_MSH6/PMS2* carriers observed for 8257 years (*p* > 0.1). We concluded that there were no differences across these groups of *path_MMR* carriers but also that this may be a type 2 error due to low numbers.

## 4. Discussion

We confirmed our hypotheses that both melanoma and lung cancer were more common among *path_MSH2* carriers than among path_*MLH1/MSH6/PMS2* carriers. These findings add to and substantiate our previous conclusion that there are clear differences among the four Lynch syndromes [1].

In addition, we demonstrated that melanoma was less common in *path_MLH1/MSH6/PMS2* carriers than in the population and that lung cancer was less common in all *path_MMR* carriers than in the population.

Our prospective observation of lower incidence of lung cancer in *path_MMR* carriers is consistent with a previous report demonstrating that, among *path_MMR* carriers, former smokers, short-term smokers, and light smokers are at reduced risk of CRC [22]. Furthermore, in the Harvard Nurses’ Health and Health Professionals Follow-up Studies, the association of pack-years smoked with CRC incidence differed by tumour mutational burden and neoantigen load [23]. Tumours with a high mutational burden (≥10 mutations/megabase) showed, compared with never-smokers, hazard ratios of 1.28 (95% CI 0.72–2.28) and 2.56 (1.61–4.07) for 1–19 and ≥20 pack-years, respectively. The researchers concluded that the immunosuppressive impact of heavy smoking could explain their findings. The corollary is that lower levels of smoking induce mutational damage sufficient to provoke immunogenicity via abnormal peptides (neo-antigens) without suppressing the immune response.

We conclude that neither melanoma nor lung cancer is part of the Lynch syndromes. In contrast, sarcoma and brain tumours have higher incidences in *path_MMR* carriers and are part of the Lynch syndromes. As with sarcoma, brain tumours, and Muir–Torre syndrome, most affected individuals with melanoma and lung cancer were *path_MSH2* carriers.

Different lifestyle factors associated with a specific *path_MMR* variant cannot explain the results: the current findings of different probabilities of developing melanoma and lung cancer that depend on which *path_MMR* is affected are novelties not previously reported. The reduced incidence of lung cancer in the older carriers is not explainable by lower rates of smoking many decades before they were aware of their cancer risks and subjected to screening. We note that the absence of smoking data is, nonetheless, a weakness in our analysis.

At first glance, one may think that MMRd tumours in endoderm-derived tissues in *path_MMR* carriers would involve carcinogenic processes that are different from those of melanoma, which arises in ectoderm-derived tissues and has a known causal environmental exposure. However, they share two properties of interest: overdiagnosis associated with screening [24,25] and the efficacy of immunotherapy [18,19]; each of these is consistent with the ability of the host immune system to identify and eliminate malignant cells in both melanoma and lung cancer. It is relevant that lung cancers are derived from endodermal cells. Our current study may, for the first time, demonstrate that lung cancer is less frequent in the Lynch syndromes and explain why this is so.

Assuming melanomas and lung cancers in *path_MMR* carriers are MMRd, we suggest that our findings indicate that the immune system may eliminate or prevent melanomas and lung cancer cells in *path_MLH1/MSH6/PMS2* carriers more readily than in non-carriers, and that this is because they are immunogenic by two different mechanisms, the second of which is MMRd and the resulting neoantigens. *Path_MLH1/MSH6/PMS2* carriers have fewer osteosarcomas, brain tumours, and Muir–Torre neoplasms than *path_MSH2* carriers, but all *path_MMR* carriers have brain tumours more frequently than the general population. In sum, the *path_MMR* variants may be anti-carcinogenic via induced immunogenicity, as well as carcinogenic; the balance between which differs across genetic variants and different tissues. The low incidence of lung cancer in *path_MMR* carriers plausibly reflects that the protective immunogenic mechanism overrides MMRd as a carcinogenic mechanism in lung epithelium.

It will be difficult to establish in humans that the combination of two different mechanisms increases the likelihood that the immune system eliminates tumours because such tumours are, by definition, no longer available for examination. It could, however, be explored in vitro or in appropriate animal models.

As a group, carriers of *path_MLH1/MSH6/PMS2* variants had a lower incidence of both melanoma and lung cancer than *path_MSH2* carriers, indicating that MMRd tumours caused by *MLH1/MSH6/PMS2* variants may have a higher probability of being eliminated or prevented by the immune system. Numbers of *path_MLH1/MSH6/PMS2* carriers having melanoma or lung cancer were insufficient to establish differences among them.

A recent report showed: (i) that increased expression of Programmed Death Ligand 1 (PD-L1) is observed with loss of *MLH1*, *MSH2*, and *PMS2*, but not with loss of *MSH6*; and (ii) that there was a previously unknown role for MSH6 as a direct regulator of PD-L1 transcription [26]. This provides a tentative step toward identifying possible differences in cancer-cell immunogenicity downstream from different *path_MMR* variants.

Any unforeseen finding should be confirmed by further study. We have begun to address this need by demonstrating similar patterns of incidence in the original and current versions of the PLSD dataset: the numbers and observation years tripled but the findings remained stable, showing that they were not due to random variation within the PLSD and are thus reasonably interpreted as a valid finding. We welcome additional studies aiming at validating or refuting our findings.

The discrepancy between the frequency of cancers among *path_MSH2* carriers versus *path_MLH1/MSH6/PMS2* carriers prior to inclusion for follow-up on the one hand and incident findings during follow-up on the other highlights, again, that neither prevalent nor retrospective findings provide accurate clinical-risk prediction. Using, for clinical purposes, personal information on previous disease, as demonstrated by this retrospective comparison, or family information (previously reported to have low positive- as well as negative-predictive values [27]) should be carefully reconsidered.

The starting point for our study was a recent report on uveal melanoma in Lynch syndrome [13], which may differ in its etiology and genetics from melanomas in general. Our results do not rule out the possibility that uveal melanomas may be more frequent in *path_MMR* carriers. The Muir–Torre syndrome could not be considered in our study because sebaceous gland adenomas were not recorded in the PLSD, and sebaceous gland and other skin cancers are not specified in the first three ICD9 positions recorded in the PLSD.

In sum, the former and current PLSD epidemiological reports, together with reports on tumour biology by others (including one on disturbed Wnt/Ctnnb1 pathways causing CRC [28] published after this work was completed) indicate that mechanisms driving carcinogenesis, including immune responses, cause interacting stochastic events that continuously alter positive and negative selection of subclones across time.

## 5. Conclusions

Our results include novel findings that also lead to a new interpretation of previous findings: (i) neither melanoma nor lung cancer is part of the Lynch syndromes; (ii) this may be explained by such tumours, if MMR proficient, being immunogenic and prone to being eliminated by the immune system, and this being even more likely if they are MMRd; (iii) the probability for such elimination depends on which MMR gene caused the MMRd; (iv) the different abilities of the pathogenic variants to trigger immune-related elimination of tumours is a property additional to their recognized different abilities to cause tumours. The current and previously reported PLSD findings, together with results recently presented by others, point to specific and additive mechanisms that both make tumours immunogenic and may trigger their elimination by the immune system. These findings may contribute to insights into ways to interrupt, simultaneously, multiple carcinogenic mechanisms to prevent or treat cancers.

## Data Availability

The original contributions presented in this study are included in the article. Further inquiries can be directed to PM. Results are categorized data extracted by the published PLSD method [14].

## Supplementary Materials

The following supporting information can be downloaded at: https://www.mdpi.com/article/10.3390/cancers18132177/s1, File S1.
