# Supplementary material for: Incidence Rates of Melanoma and Lung Cancer Are Generally Low in the Lynch Syndromes and Vary Across path_MMR Variants: A Prospective Lynch Syndrome Database Report
Source: Cancers (Basel). 2026 Jul 7;18(13):2177. doi: 10.3390/cancers18132177 (PMC13360418; doi:10.3390/cancers18132177)
Supplement: Supplementary file 1 [file cancers-18-02177-s001.zip › cancers-4370149-supplementary.pdf]

PLSD collaborators:

Lone Sunde (Department of Clinical Genetics, Aalborg University Hospital, Aalborg, Denmark,Department of Clinical Medicine, Aalborg University, Gistrup, Denmark, The Danish HNPCC Register, Gastro Unit, Copenhagen University Hospital - Amager and Hvidovre, Copenhagen, Denmark); Karin A. W. Wadt (Department of Clinical Genetics, Rigshospitalet, Denmark);Mark A Jenkins (Melbourne School of Population and Global Health, Centre for Epidemiology and Biostatistics, The University of Melbourne, Parkville, Australia); Christoph Engel (Institute for Medical Informatics, Statistics and Epidemiol-ogy, University of Leipzig, 04107, Leipzig, Germany); Stefan Aretz (Institute of Human Genetics, National Center for Hereditary Tumor Syndromes, Medical Faculty, University Hospital Bonn, University of Bonn, Bonn, Germany);Maartje Nielsen (Department of Clinical Genetics, Leids Universitair Medisch Centrum, Leiden, The Netherlands); Sanne W. Bajwa-ten Broeke (Affiliation: Department of Genetics, University Medical Center Groningen, Groningen, the Netherlands);Dafydd Gareth Evans (Manchester Centre for Genomic Medicine, Manchester University NHS Foundation Trust, Manchester, UK); John Burn (Faculty of Medical Sciences, Newcastle University, Newcastle Upon Tyne, UK) ;Elke Holinski-Feder (Campus Innenstadt, Medizinische Klinik und Poliklinik IV, Klinikum der Universität München, Munich, Germany; Center of Medical Genetics, Munich, Germany) ; Bernardo Bonanni (Division of Cancer Prevention and Genetics, IEO, European Institute of Oncology IRCCS, Milan, Italy) ;Lucio Bertario (Division of Cancer Prevention and Genet-ics, IEO, European Institute of Oncology IRCCS, Milan, Italy) ;Monica Marabelli (Division of Cancer Prevention and Genetics, IEO, European Institute of Oncology IRCCS, Milan, Italy) ;Serrano Davide (Division of Cancer Prevention and Genetics, IEO, European Insti-tute of Oncology IRCCS, Milan, Italy) ;Zohar Levi (Service High Risk GI Cancer Gastro-enterology, Department Rabin Medical Center, Israel); Ingrid Winship (Colorectal Medi-cine and Genetics, The Royal Melbourne Hospital, Melbourne, Australia; Department of Medicine, Melbourne University, Melbourne, Australia; Department of Medicine, Uni-versity of Melbourne, Melbourne, Australia); Luigi Laghi (Laboratory of Molecular Gastroenterology, IRCCS Humanitas Research Hospital, Rozzano, Italy; Department of Medicine and Surgery, University of Parma, Parma, Italy) ; Florencia Neffa (Hospital Fuerzas Armadas, Grupo Colaborativo Uruguayo, Investigación de Afecciones Onco-lógicas Hereditarias, Montevideo, Uruguay); Adriana Della Valle (Hospital Fuerzas Ar-madas, Grupo Colaborativo Uruguayo, Investigación de Afecciones Oncológicas Hered-itarias, Montevideo, Uruguay); Karl Heinimann (Medical Genetics, Institute for Medical Genetics and Pathology, University Hospital Basel, Basel, Switzerland); Tadeusz Dębniak (Department of Genetics and Pathology, International Hereditary Cancer Center, Szczecin, Poland); Robert Fruscio (UO Gynecology, Fondazione IRCCS San Gerardo dei Tintori, Monza, Italy; Department of Medicine and Surgery, University of Milan-Bicocca, Milan, Italy); Karin Alvarez-Valenzuela (Clínica Universidad de los Andes, Santiago, Chile; Programa Cáncer Heredo Familiar, Santiago, Chile); Francisco López Köstner (Clínica Universidad de los Andes, Chile. Programa Cáncer Heredo Familiar, Santiago, Chile); Lior H Katz (Department of Gastroenterology, Hadassah Medical Center, Faculty of Medicine, Hebrew University of Jerusalem, Israel); Ido Laish (Department of Gastroenterology, Hadassah Medical Center, Faculty of Medicine, Hebrew University of Jerusalem, Isra-el); Elez Vainer (Hadassah Medical Center, Israel); Carlos Vaccaro (Hereditary Cancer Program (PROCANHE), Hospital Italiano de Buenos Aires, Ciudad Autónoma de Buenos Aires, Argentina); Dirce Maria Carraro (Clinical and Functional Genomics Group, A.C.Camargo Cancer Center, Sao Paulo, Brazil); Kevin Monahan (Lynch Syndrome & Family Cancer Clinic, St Mark's Hospital, London, UK); Elizabeth Half (Gastrointestinal Cancer Prevention Unit, Gastroenterology Department, Rambam Health Care Campus, Haifa, Israel); Aine Stakelum (St Vincent's University Hospital, Ireland); D. Winter (St Vincent's University Hospital, Ireland) ; Rory Kennelly (St Vincent's University Hospital, Ireland); Nathan Gluck (Department of Gastroenterology, Tel-Aviv Sourasky Medical Center and Sackler Faculty of Medicine, Tel-Aviv University, Tel-Aviv, Israel); Harsh Sheth (Foundation for Research in Genetics and Endocrinology, Institute of Human Ge-netics, Ahmedabad, India); Naim Abu-Freha (Soroka University Medical Center, Ben-Gurion University of the Negev, Beer Sheva,

Israel); Benedito Mauro Rossi (Hospital Sirio Libanes, Sao Paulo, Brazil); Giulia Martina Cavestro (Gastroenterology and Gas-trointestinal Endoscopy Unit, Division of Experimental Oncology, IRCCS San Raffaele Scientific Institute, Vita-Salute San Raffaele University, Milan, Italy); Mannucci Alessandro (Gastroenterology and Gastrointestinal Endoscopy Unit, Division of Experimental Oncology, IRCCS San Raffaele Scientific Institute, Vita-Salute San Raffaele University, Milan, Italy); Leonardo S Lino-Silva (Surgical Pathology, Instituto Nacional de Cancero-logia, Mexico City, Mexico); Karoline Horisberger (Department of Surgery, Universi-tätsmedizin Mainz, Mainz, Germany); Huw Thomas (St Mark's Hospital, Department of Surgery and Cancer, Imperial College London, London, UK.); Norma Teresa Rossi (Fundación para el Progreso de la Medicina, Sanatorio Allende, Córdoba, Argenti-na); Leandro Apolinário da Silva (Hospital Universitário Oswaldo Cruz, Universidade de Pernambuco, Recife, Brazil; SEQUIPE, Recife, Brazil); Attila Zaránd (St John Central Hospital, Budapest, Hungary); Laura E Thomas (Institute of Life Science 1, Swansea University, Swansea, SA28PP, UK.); Jukka-Pekka Mecklin (Faculty of Sport and Health Sciences, University of Jyväskylä, Jyväskylä, Finland; Department of Education and Re-search, The Wellbeing Services of Central Finland, Jyväskylä, Finland); Kirsi Pylvänäinen (Department of Education and Science, The Wellbeing Services of Central Finland, Jyväskylä, Finland); Laura Renkonen-Sinisalo (Applied Tumor Genomics Research Pro-gram, Research Programs Unit, University of Helsinki, Helsinki, Finland; Department of Abdominal Surgery, Helsinki University Hospital, Helsinki, Finland); Anna Lepistö (Ap-plied Tumor Genomics Research Program, Research Programs Unit, University of Helsinki, Helsinki, Finland; Department of Abdominal Surgery, Helsinki University Hospital, Helsinki, Finland); Polly A Newcomb (Public Health Sciences Division, Fred Hutchinson Cancer Research Center, Seattle, WA, USA); Loïc Le Marchand (University of Hawaii Cancer Center, Honolulu, HI, USA); Verena Steinke-Lange (Medizinische Klinik und Poliklinik IV, Campus Innenstadt, Klinikum der Universität München, Munich, Germany; MGZ-Medical Genetics Center, Munich, Germany); Deepak Vangala (Department of Medicine, Knappschaftskrankenhaus, Ruhr-University Bochum, Bochum, Germany); Silke Zachariae (Institute for Medical Informatics, Statistics and Epidemiology, Leipzig Uni-versity, Leipzig, Germany); Diana Le Duc (Institute for Clinical Genetics, University Hospital Carl Gustav Carus at TUD Dresden University of Technology and Faculty of Medicine of TUD Dresden University of Technology, Dresden, Germany); Marcus Franke (Institute for Clinical Genetics, University Hospital Carl Gustav Carus at TUD Dresden University of Technology and Faculty of Medicine of TUD Dresden University of Tech-nology, Dresden, Germany); Katrin van Beekum (Department of Internal Medicine I, University Hospital Bonn; National Center for Hereditary Tumor Syndromes, University Hospital Bonn, Bonn, Germany); Barbara Klink (Arbeitsgruppe erbliche gastrointestinale Tumore, Medizinische Klinik und Poliklinik IV, Campus Innenstadt, Klinikum der Uni-versität München, München, Germany); Hoa HP Nguyen (Department of Human Genetics, Ruhr-University Bochum, Bochum, Germany); Michael Pohl (Department of Medicine, Universitätsklinikum Knappschaftskrankenhaus Bochum GmbH, Ruhr-University Bochum, Bochum, Germany); Jane Figueiredo (Public Health Sciences Division, Fred Hutchinson Cancer Research Center, Seattle, WA, USA); Revital Kariv (Shaare Zedek Medical Hospital, Jerusalem, Israel); G. Rosner (St Vincent's University Hospital, Ire-land); Robert Hüneburg (Department of Internal Medicine I, University Hospital Bonn; National Center for Hereditary Tumor Syndromes, University Hospital Bonn, Bonn, Germany), Silke Redler (Institute for Medical Informatics, Statistics and Epidemiology, Leipzig University, Leipsig, Germany; Institute of Human Genetics, Medical Faculty and University Hospital Düsseldorf, Heinrich-Heine-University Düsseldorf, Düsseldorf, Germany); Reinhard Büttner (Institute of Pathology, Faculty of Medicine and University Hospital Cologne, Cologne, Germany); Fiona Lalloo (Manchester Centre for Genomic Medicine, Manchester University NHS Foundation Trust, Manchester, UK); Emma J. Da-vidson (Gynaecological Oncology Research Group, Manchester University NHS Founda-tion Trust, Manchester, UK; Division of Cancer Sciences, University of Manchester, Manchester, UK); Miriam Mints (Division of Obstetrics and Gynaecology, Department of Women's and Children's Health, Karolinska Institutet, Karolinska University Hospital, Stockholm, Sweden); Douglas Tjandra (Colorectal Medicine and Genetics, The

Royal Melbourne Hospital, Melbourne, Australia; Department of Medicine, Melbourne Univer-sity, Melbourne, Australia); Patricia Esperon (Laboratory of Molecular Gastroenterology, IRCCS Humanitas Research Hospital, Rozzano, Italy; Department of Medicine and Sur-gery, University of Parma, Parma, Italy); Walter Pavicic (Instituto de Medicina Traslacional e Ingenieria Biomedica (IMTIB), CONICET IU, Hospital Italiano de Buenos Aires, Buenos Aires, Argentina); Gabriela Möslein (Surgical Center for Hereditary Tumors, Ev. Bethesda Khs Duisburg, University Witten-Herdecke, Herdecke, Germany).
